# Preparation and Characterization of Lignin Nanoparticles from Different Plant Sources

**DOI:** 10.3390/polym16111610

**Published:** 2024-06-06

**Authors:** Isidora Ortega-Sanhueza, Victor Girard, Isabelle Ziegler-Devin, Hubert Chapuis, Nicolas Brosse, Francisca Valenzuela, Aparna Banerjee, Cecilia Fuentealba, Gustavo Cabrera-Barjas, Camilo Torres, Alejando Méndez, César Segovia, Miguel Pereira

**Affiliations:** 1 Facultad de Ciencias Forestales, Universidad de Concepción, Concepción 4070374, Chile; camiltorres@udec.cl (C.T.); amendez@udec.cl (A.M.); 2 Laboratoire d’Etude et de Recherche sur le MAtériau Bois (LERMAB), Faculté des Sciences et Techniques, Université de Lorraine, 54500 Vandœuvre-lès-Nancy, France; victor.girard@univ-lorraine.fr (V.G.); isabelle.ziegler@univ-lorraine.fr (I.Z.-D.); hubert.chapuis@univ-lorraine.fr (H.C.); nicolas.brosse@univ-lorraine.fr (N.B.); 3 Instituto de Ciencias Aplicadas, Facultad de Ingeniería, Universidad Autónoma de Chile, Talca 3467987, Chile; franci.valenzuela@hotmail.com (F.V.); aparna.banerjee@uautonoma.cl (A.B.); 4 Unidad de Desarrollo Tecnológico (UDT), Universidad de Concepción, Av. Cordillera 2634, Parque Industrial Coronel, P.O. Box 4051 Mail 3, Concepción, Chile; c.fuentealba@udt.cl; 5 Centro Nacional de Excelencia para la Industria de la Madera (CENAMAD), Pontificia Universidad Católica de Chile, Av. Vicuña Mackena, 4860, Santiago 7820436, Chile; 6 Facultad de Ciencias para el Cuidado de la Salud, Universidad San Sebastián Campus Las Tres Pascualas, Lientur 1457, Concepción 4080871, Chile; gustavo.cabrera@uss.cl; 7 Centre d’Essais Textile Lorrain, CETELOR—Université de Lorraine, 27 rue Philippe Seguin, 88051 Epinal, France; cesar.segovia@univ-lorraine.fr; 8 Facultad de Ingeniería, Departamento de Ingeniería Química, Universidad de Concepción, Concepción 4070374, Chile

**Keywords:** lignin sources, nanoparticle preparation, antioxidant activity, characterization

## Abstract

This article presents new research on producing lignin nanoparticles (LNPs) using the antisolvent nanoprecipitation method. Acetone (90%) served as the lignin solvent and water (100%) as the antisolvent, using five types of lignins from various sources. Comprehensive characterization techniques, including NMR, GPC, FTIR, TEM, and DLS, were employed to assess both lignin and LNP properties. The antioxidant activity of the LNPs was evaluated as well. The results demonstrated the successful formation of spherical nanoparticles below 100 nm with initial lignin concentrations of 1 and 2%^w^/_v_. The study highlighted the crucial role of lignin purity in LNP formation and colloidal stability, noting that residual carbohydrates adversely affect efficiency. This method offers a straightforward, environmentally friendly approach using cost-effective solvents, applicable to diverse lignin sources. The innovation of this study lies in its demonstration of a cost-effective and eco-friendly method to produce stable, nanometric-sized spherical LNPs. These LNPs have significant potential as reinforcement materials due to their reinforcing capability, hydrophilicity, and UV absorption. This work underscores the importance of starting material purity for optimizing the process and achieving the desired nanometric dimensions, marking a pioneering advancement in lignin-based nanomaterials.

## 1. Introduction

Lignin is the most abundant aromatic biopolymer on Earth [1,2]. It is an irregular, three-dimensional macromolecule that is biodegradable, non-toxic, and hydrophobic in nature [3]. Lignin is composed of p-hydroxyphenyl (H), guaiacyl (G), and syringyl (S) units that derive from the polymerization of the hydroxycinnamyl alcohols, p-coumaryl, coniferyl, and sinapyl alcohol, respectively. Units are linked by numerous types of bonds, with the β-*O*-4 aryl ether bonds being the most prevalent (around 50%) [4]. In hardwood trees, lignin primarily consists of S and G units, while in softwoods, the predominant units are G units [5]. The main function of lignin is to bind fibers together, providing rigidity, and to protect trees against chemical and physical attacks. Lignin acts as a matrix surrounding cellulose and hemicellulose, with the three biopolymers forming a closed, highly interconnected network [6], with lignin accounting for approximately 25% of biomass [7].

Lignin has gained prominence as a low-cost byproduct of the pulp and paper industry [8], where 50 million tons are generated annually. Its primary use is energy generation, with only 5% utilized for biorefinery applications in the food, cosmetics, pharmaceuticals, chemicals, agriculture, and textiles sectors [3]. The valorization of lignin is also associated with its chemically stable and complex structure, which offers potential for antioxidant and antimicrobial properties, high thermal stability, and UV protection [9,10], providing a wide range of applications and research potential.

However, the use of lignin as a high-value-added product is limited due to its large particle size, poor dispersibility, color, and irregular morphology, which are present in different biomass species [11]. To overcome these limitations, various alternatives can be employed, such as using chemical reactions to modify lignin. Its abundance of available functional groups enables surface functionalization through chemical reactions such as esterification, carboxymethylation, hydroxymethylation, sulfonation, and oxidation [4]. Additionally, the production of nanoscale materials based on lignin offers another avenue [12].

Due to recent research focused on nanotechnology, lignin-based nanomaterials have attracted industrial and academic attention [13]. Manipulating lignin into micro or nanostructures can offer a variety of uses since feasible and adjustable lignin nanoparticle (LNP) synthesis exists according to its applications. LNPs exhibit reinforcing properties, UV absorption, antifungal, antibacterial, and antioxidant properties, with high thermal stability, biodegradability [10], and flame-retardant properties [8].

However, there is no consensus on the best production process, as different extraction techniques yield LNPs with different characteristics and uses; some of the techniques used include acid precipitation, instant precipitation, CO_2_ precipitation, sonication, solvent exchange precipitation or nanoprecipitation, dialysis, aerosol flow, and microemulsions, among others [14]. Additionally, there are different methods for producing micro or nanoparticles, but not all particles result in spherical shapes, and spherical LNPs are more functional [15]. Each method presents advantages and disadvantages, so combinations of techniques have been used to maximize results. Below are descriptions of some of the most commonly used processes. Aerosol flow reactor: Diluted lignin solutions undergo controlled drying, producing dry particles of a wide size range. This process is suitable for all types of lignin, provided the appropriate solvent is used. The most commonly used solvents are water, alcohols, acetone, and DMF [15]. Acid precipitation: This is one of the most well-known and widely used processes, producing irregular network structural formations and unstable sedimentation due to the protonation of lignin’s charged groups, which affects LNP stability. The most commonly used acids are HCl, HNO_3_, or H_2_SO_4_ [16]. Instant precipitation: This precipitation is induced by a pH drop in an ethylene glycol solution to sinter the nanoparticles. This pH drop causes lignin supersaturation, followed by nucleation and particle growth. Parameters controlling particle formation are: (a) initial lignin concentration, (b) acid molarity, and (c) amount of acid added. The particles are diluted with water to halt further size increases and then characterized [17]. CO_2_ precipitation: This method employs CO_2_ as an antisolvent through crystallization and nucleation. The characteristics of these LNPs can be controlled by adjusting temperature, pressure, CO_2_ flow, and lignin concentration [18].

In this study, the method used is antisolvent precipitation. This process generally involves dissolving lignin in a water-miscible organic solvent and precipitating it in a precipitating agent (usually water). During this process, a self-assembly mechanism governs nanoparticle formation due to lignin hydrophobic and hydrophilic interactions to reduce the contact area with the precipitating agent, thus stimulating nano-level aggregate formation [11]. The produced LNPs are synthesized from a renewable source and have potential applications in various fields. For instance, in agriculture, they can aid in resilience and sustainability by serving as nano-carriers for environmentally sustainable pesticides [9]. They can also be used as ultraviolet-absorbing products to improve their properties and durability, flame retardancy, and medical and drug delivery systems [19].

Other applications for LNPs include their use as dispersants, primarily in spherical LNPs, employed as surfactants, plasticizers, or emulsifiers depending on the field of application, as they enable the mixing of immiscible liquid phases and improve particle suspension stability [15].

Moreover, given lignin’s intrinsic nature (binding fibers in the cell wall), one of its most logical uses is in adhesives. Lignin has been tested as a partial replacement for toxic phenol-formaldehyde resins. However, incorporating lignin can affect board performance in some mechanical properties, with substitution values exceeding 40% [20]. Nevertheless, it has been reported that spherical LNPs could be of interest in the biomedical adhesive area to aid in bonding soft tissue, especially when complemented with hydrogels [12]. In the biomedical field, LNPs can also be used for drug administration and transport [21]. LNPs can be employed for the preparation of emulsions and colloids for sunscreens due to their UV absorption capacity [22]. Furthermore, LNPs can participate in chemical bonding due to the wide variety of their polar functional groups, allowing them to be used for the adsorption of various pollutants and heavy metals in wastewater [23,24]. Lignin reactivity is influenced by its complex molecular structure and steric hindrance. Unmodified (native) lignin contains phenolic and aliphatic hydroxyl groups, non-condensed guaiacyl groups, and carbonyl groups, which can promote its insertion into bio-based thermoset and thermoplastic polymers, potentially reducing the use of synthetic polymers [25].

One of the significant advantages of LNPs is their smaller size compared to lignin (macro), allowing better interaction with polymeric matrices, and their ability to enhance compound properties [26]. Spherically shaped LNPs can disperse well in water and ensure the formation of a long-lasting and homogeneous dispersion without chemical treatments [27]. Thus, LNPs can be used as additives in polymeric matrices such as rubber [28], polyvinyl alcohol (PVA) [10], epoxy, and thermoplastics [29], among others. The applications and research potential of LNPs are extensive and promising, but the systematic production of lignin nanoparticles is a challenging process due to the molecular weight, chemical, and physical properties of lignin, which highly depend on the source and extraction methods. The combined impact of the fractionation and extraction processes on lignin properties is even more dominant than the effect of the lignin origin source. This work aims to clarify the impact of lignin from different sources and extraction methods on the production and characterization of LNPs, obtained through the method of antisolvent precipitation [30]. The LNPs characterizations in this work intend to discover potential applications as UV protectors, fungi resistance, and vapor barriers in films and coatings.

## 2. Materials and Methods

### 2.1. Reagents

To produce the LNPs, acetone for analysis from Merck (Nancy, France) (100014) and filtered and purified water from the Milli-Q Plus water purification system (Nancy, France) were used.

### 2.2. Isolation of Lignin from Different Sources

Five lignins and LNPs (Table 1) from different sources were used in the present investigation. Lignin from *Eucalyptus globulus*, *Pinus radiata* D. Don, and *Triticum* spp. (wheat straw) was obtained at a pilot scale and extracted using an organosolv process with acetic acid as a solvent medium, as mentioned by Berg [31]. Another kind of lignin studied corresponded to a commercial Kraft lignin of high purity obtained from softwood species of *Picea abies* and *Pinus sylvestris*. The last lignin was obtained from the bark of *Eucalyptus globulus* using an organosolv process with ethanol and acid precipitation.

### 2.3. Lignin Characterization

#### 2.3.1. Biomass Analysis: Lignin and Sugars in Liquid Fraction

The purity of each lignin was determined using National Renewable Energy Laboratory (NREL) labeled protocols and TAPPI method T222 [32]. The determination of insoluble lignin (purity) was carried out by gravimetry. High-Performance Anion Exchange Chromatography with Pulsed Amperometry Detection (HPAE-PAD, ICS-3000 Dionex™, Nancy, France) and a Dionex™ CarboPac PA-20 (3 × 150 mm) analytical column was used to analyze the monomeric sugars in liquid fractions. Monosaccharides were eluted at 35 °C with a flow rate of 0.4 mL/min according to the following composition: 99.2% ultrapure water/250 mM NaOH 0.8%: 0–20 min; 75% ultrapure water/250 mM NaOH 20%/NaOAc (1 M)–NaOH (20 mM) 5% 20–37 min; 40% ultrapure water/250 mM NaOH 20%/NaOAc (1 M)–NaOH (20 mM) 40% 37–41 min. Washing and the necessary equilibration time were performed after each elution. The determination of fucose, arabinose, rhamnose, galactose, glucose, xylose, mannose, galacturonic acid, and glucuronic acid was carried out by external calibration with standards.

#### 2.3.2. Antioxidant Activity

The antioxidant activity of lignin was determined using the DPPH radical scavenging method. This method relies on the reduction of DPPH by adding a radical species or an antioxidant, which decolorizes the DPPH solution. Antioxidant activity is measured by the decrease in absorption at 520 nm using a Shimatzu UV-1900i spectrophotometer (Nancy, France). Using an initial solution (A) of DPPH of 20 mg/mL in ETOH, which is diluted to 50 mg/L (solution B), in parallel, a solution (C) of 10 mg of lignin in 5 mL of ETOH is prepared for the measurement. Six solutions (D) are made with different aliquots of solution C (10, 25, 50.75, 125, and 200 µL) in a 5 mL volumetric flask with solution (B). Solutions (D) are shaken for exactly 1 h before being measured in the spectrophotometer.

#### 2.3.3. Analysis of Molecular Weight Distribution (by GPC)

To determine the distribution and average molecular weight values of the lignins, size exclusion chromatography (SEC) was performed. For the analysis, the lignin sample is dissolved in NaOH (10 mM and 5 mg/mL) and stirred for 24 h, then it is filtered with a 0.45 µm PTFE filter and 20 µL is injected into a chromatograph Shimadzu Prominence™ with a UV detector at 280 and 254 nm, and a combination of Phenomenex PolySep-SEC GFC-P 2000 and PolySep-SEC GFC-P 3000 (Nancy, France) were used for the analyses. The separation was performed at 35 °C and eluted with NaOH (10 mM, 0.4 mL/min). Finally, the calibration curve was plotted using Agilent Technologies™ GPC/SEC calibration kits (Nancy, France) for pullulans.

#### 2.3.4. Quantitative NMR Spectroscopy of Lignin

Lignin structure analysis was conducted using Heteronuclear Single Quantum Coherence (HSQC) Nuclear Magnetic Resonance (NMR). In summary, 100 mg of purified and dried Lignin were dissolved in 500 µL of dimethyl sulfoxide-d6 (DMSO-d6, 99.8%) for the 13C-1H HSQC analysis. Spectra were acquired using a Bruker™ Avance III 400 MHz spectrometer at 50 °C with a relaxation delay of 25 s (Figures S1–S10). Using the following formulas for the quantification (%) of substructures in lignin [33].

Total aromatic = (((S2/6 + S’2/6)/2) + Scondensed) + ((G2 + G5 + G6 − H2/6)/3) + (H2/6/2)

Ratio S = (((S2/6 + S’2/6)/2) + Scondensed): total aromatic × 100%

Ratio G = ((G2 + G5 + G6 − H2/6)/3): total aromatic × 100%

Ratio H = (H2/6/2): total aromatic × 100%

β-*O*-4 linkages = (β-*O*-4α + β′-*O*-4 α)/total aromatic × 100

β-5 linkages = β-5α/total aromatic × 100

β-β linkages = β-βα/total aromatic × 100

#### 2.3.5. Thermogravimetric Analysis: TGA and DSC

Thermogravimetric analysis (TGA) was performed in a Termobalance Netzsch STA409PC under a nitrogen atmosphere (50 L/min). Samples (30 mg) were heated from 25 to 600 °C at a heating rate of 5 °C/min.

Differential scanning calorimetry (DSC) analysis was performed using DSC 4000 (Perkin Elmer) equipment under an N2 atmosphere (50 L/min). The samples (10 mg) were heated from 25 to 350 °C at a heating rate of 5 °C/min.

#### 2.3.6. FTIR Analysis

The FT-IR spectra were acquired using a Fourier-transform infrared spectrometer (Perkin Elmer FTIR/NIR spectrometer, Frontier). A total of 32 scans were taken for each sample in a spectral range of 4000–650 cm^−1^ with a resolution of 4 cm^−1^. All spectra were acquired and processed using OriginPro8 software.

### 2.4. LNP Preparation and Characterization

#### 2.4.1. Lignin Nanoparticle Preparation

LNPs were prepared using the antisolvent precipitation method. Firstly, solutions with the five characterized lignins at concentrations of 1%, 2%, and 5%^w^/_v_ in 90%^v^/_v_ aqueous acetone were prepared, and then placed in an ultrasonic bath for one hour to completely dissolve the lignin. To produce LNPs, 10 mL of each lignin solution was injected quickly into 90 mL of Milli-Q water to obtain 100 mL of LNP for each lignin and initial concentration.

#### 2.4.2. Dynamic Light Scattering (DLS) and Zeta Potential (ζ)

The size distribution, size average, polydispersity index (PI), and ζ-potential of prepared suspensions were determined using a Malvern™ Zetasizer Nano ZS instrument (Nancy, France). Measurements were performed immediately after precipitation. Size distribution, size average, and PI were determined by conducting Multi-Angle Dynamic Light Scattering (MADLS) analysis on triplicate samples. While many studies utilize a back angle (173°) to minimize particle size, this may compromise size distribution accuracy compared to MADLS, which integrates measurements from three angles. For long-term stability tests, suspensions were stored at 8 °C and reheated to 25 °C prior to measurements. ζ-potential analyses were conducted using the same instrument, employing special folded capillary zeta cells (DTS 1070) at 25 °C.

#### 2.4.3. Transmission Electron Microscopy (TEM) and Estimation of Diameter

The samples were analyzed in the Electron Microscopy Laboratory of the University of Santiago de Chile (USACH) on the Hitachi H7700 equipment (Santiago, Chile). High-resolution images of lignin nanoparticle samples were collected using Transmission Electron Microscopy (TEM) images. Given the large number of particles, an automated method of measurement was employed. For this task, the Ultralytics YOLOv8 object detection model was utilized to identify and locate LNPs in the images. This model is known for its high speed and accuracy in object detection. For each detected nanoparticle, bounding boxes were generated. These boxes were adjusted to closely match the dimensions of each nanoparticle, allowing for precise delineation of the region of interest. Utilizing the confidence parameter of the model, a threshold was set to filter out incorrect predictions.

Using the coordinates of the bounding boxes and the TEM image resolution, the diameters of the nanoparticles were calculated by measuring the maximum distance between opposite edges within each bounding box on the *X* and *Y* axes, and averaging them, providing an estimate of the particle’s diameter in pixel units. By using the indicated scale and its pixel transformation, each measurement was converted into a metric scale to obtain a distribution of particle sizes across the samples.

#### 2.4.4. Determination of Antioxidant Capacity

##### Scavenging Activity of H_2_O_2_

The radical scavenging activity of hydrogen peroxide (H_2_O_2_) was determined in various samples of lignin nanoparticles N-LE, N-LP, N-LC, and N-LEB (0.19% solid concentration) following Ruch, Cheng, and Klaunig [34] after some modifications, along with control ascorbic acid (1 mM). Briefly, a volume of 50 μL of LNPs sample was taken to record the absorbance at 230 nm (A_2_) using the Mobi-Microplate spectrophotometer (μ2 MicroDigital, MOBI, Seoul, Republic of Korea). Afterwards, 120 μL of 0.1 M phosphate buffer (pH 7.40) and 30 μL of H_2_O_2_ at 30%^w^/_v_ were mixed with it. The mixture was then incubated for 10 min at 30 °C, and again, the absorbance (A_1_) of the reaction mixture was recorded at 230 nm, followed by the calculation of the •OH scavenging activity using the following equation:

[1 − (A_1_ − A_2_)/A_0_] × 100

where A_1_ is the absorbance of the sample with H_2_O_2_, A_2_ is the absorbance of the sample without H_2_O_2_, and A_0_ is the absorbance of distilled water.

##### Ferric Reducing Antioxidant Power (FRAP) Activity

The ferric reducing antioxidant power (FRAP) assay was performed using the EZAssay^TM^ antioxidant activity estimation (FRAP) kit (HiMedia Laboratories PVT Ltd.) (Talca, Chile) following the steps mentioned in the protocol. Briefly, 100 μL of aqueous suspension (0.19% solid concentration) of each lignin nanoparticle sample (N-LE, N-LP, N-LC, and N-LEB) were prepared. And 100 μL of the chromogenic substrate were added to each sample, as well as to a blank sample (1X assay buffer) and the 100 μL FeCl_2_ standard samples. Then, the reaction mixture was incubated for 10 min in the dark at room temperature. The absorbance of the reaction mixture was recorded at 560 nm using a Mobi-Microplate spectrophotometer (μ2 MicroDigital, MOBI, Seoul, Republic of Korea), and the slope of the FeCl_2_ standard curve as well as the corrected absorbance were calculated using the equation [35]. The experiment was performed in triplicate. Finally, the antioxidant concentration of each sample was calculated from the FeCl_2_ standard curve using the equations proposed by Santos [33] and Apel [36].

Corrected absorbance = Sample absorbance_(560nm)_ − 1× assay buffer absorbance_(560nm)_

$$\mathrm{Fe}(\mathrm{II})\;iron\;equivalents\;(\mu M)=\frac{\left(Corrected\;absorbance)-(Y\;intercept\right)}{Slope}$$

### 2.5. Statistical Analysis

All samples were run at least three times, and the results are expressed as means ± standard deviations. A *p*-value < 0.05 was considered statistically significant using ANOVA, with a Tukey test using SAS University Edition statistical software.

## 3. Results

### 3.1. Lignin Characterization

#### 3.1.1. Biomass Analysis: Lignin and Sugars in Liquid Fraction

The results shown in Table 2 were obtained by analyzing the five lignin samples following the TAPPI T222 methodology. Sample LC, of Kraft commercial origin, exhibits the highest purity percentage, followed by samples LP and LE from *P. radiata* and *E. globulus* acetosolv, respectively. These three samples yielded low sugar values, below 2%. For sample LEB, obtained from an organosolv process of Eucalyptus globulus bark, the lignin percentage decreases by 12% compared to the purity of LC, making it the second lowest value, comparable only to LWS, wheat straw acetosolv, which has a purity decrease of 16% compared to LC. These low values are associated with the high percentage of sugars, mainly from hemicellulose, with values up to 6%, which can be attributed to the difficulty of isolating lignin from these materials due to the higher content of extractives and low molecular weight oligosaccharides present in the bark and wheat straw samples, as well as the structural and chemical differences of their lignin compared to wood-derived lignin.

Regarding statistical analysis, each lignin quantification is statistically different from the others.

#### 3.1.2. Antioxidant Activity

It has been reported that lignin presents antioxidant properties [10] due to its rich aromatic structure. Therefore, the different functional groups and their phenolic content are the most important factors. Although the β-*O*-4 linkage is the most abundant in most lignins, this linkage has lower bond dissociation energies (BDE) (222 kJ/mol) [37], making it easier to cleave to generate new phenolic hydroxyls, which benefits antioxidant activity, in addition to a low molecular weight. Thus, antioxidant activity depends on the biomass species and the extraction method.

The DPPH assay is used to quantify the activity of free radical scavenging. The reaction is based on the decrease in color that occurs when the odd electron of the nitrogen atom in DPPH is reduced upon receiving a hydrogen atom from antioxidant compounds [38].

The DPPH assay (Table 3) shows that LE exhibits the highest antioxidant power, as it presents the lowest value of 50% inhibition concentration (IC50), defined as the amount of antioxidant required to reduce the initial concentration of DPPH by 50%. Conversely, LC has the highest IC50 value among the five lignins.

The antioxidant activity index (AAI), as proposed by Scherer and Godoy [38], shows that antioxidant activity is low when AAI < 0.5, moderate with AAI values between 0.5 and 1.0, strong with AAI values between 1.0 and 2.0, and very strong with samples with AAI values > 2.0. Following these parameters, LE is the one that presents a very strong antioxidant activity. This may be attributed to the greater amount of phenolic hydroxyl groups, fewer aliphatic hydroxyl groups, and low molecular weight, since these conditions show high antioxidant activity [39]. In the case of LP, LWS, and LEB, their values are in the second-strongest range of AAI. Finally, LC presents moderate antioxidant activity.

With respect to the results, although I was able to calculate the *p*-values for the set of measurements, they are not comparable due to being from different sources. However, the dataset shows significant differences.

#### 3.1.3. Analysis of Molecular Weight Distribution (by GPC)

The organosolv process, being a solvent extraction approach, represents an excellent method for fractionating lignin and obtaining the desired molecular weights and functional group contents [40]. The Mw results (Table 4) for the obtained samples show very close retention times, with significant differences among the values obtained (*p*-value). The value obtained for LEB stands out, as despite being obtained from an organosolv process, which should allow for obtaining lignin of lower molecular weight, as is the case with LE, LP, and LWS, this type of lignin presents the highest molecular weight. This may be attributed to its initial biomass (bark), which, given its more complex nature than wood, hinders its optimal isolation.

#### 3.1.4. Quantitative NMR Spectroscopy of Lignin

Nuclear magnetic resonance (NMR) analysis allows for the identification of the structure and quantity of lignin-carbohydrate linkages involved in lignin-carbohydrate complexes (LCC) [41]. The NMR results (Table 5) show that LE has the lowest amount of β-O-4 linkages, which can be attributed to greater depolymerization during its pretreatment, followed by LC and LP. The high amount of β-*O*-4 linkages in LWS and LEB may be due to their high polysaccharide content (Table 2), which is responsible for their low purity. The absence of β-5 linkages in LE and LEB correlates with the low initial amount of this type of linkage, given the steric hindrance of the S-type monolignol to form linkages at C5 [42]. Finally, the S/G ratio is consistent with expectations for softwood samples (LP and LC) and hardwood samples (LE and LEB), as well as for the wheat straw sample (LWS).

#### 3.1.5. Thermogravimetric Analysis (TGA and DSC)

**Figure 1:**
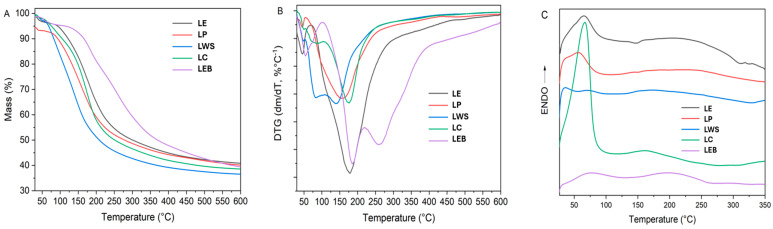
Thermogravimetric analysis of TG (**A**), DTG (**B**), and DSC (**C**) of lignin.

Thermogravimetric analysis is a technique that has been widely used for lignin characterization [43]. It allows for the determination of macromolecular thermal stability, decomposition temperature, and char yield, respectively [44]. In this work, the thermal behavior of lignin, obtained by different processes and diverse biomass, is compared. The thermogravimetric curves (TG, DTG, and DSC) are shown in Figure 1, and the analysis results are presented in Table 6.

In Figure 1A,B, it is shown that thermal decomposition of lignin occurs over a wide range of temperatures. In the first stage, at temperatures below 100 °C, the loss of unbound water occurs. This is an endothermic process (Figure 1C) that has been reported by several authors [45].

A second effect, corresponding to the lignin mayor degradation process, occurs in a broad temperature range (70–467 °C). From the TG-DTG curves, it can be observed that all lignin shows different thermal stability depending on their source or preparation process. The least thermally stable samples were LWS and LP, which have a maximum decomposition rate temperature (Tpeak) of 140 °C and 157 °C, with an associated mass loss of 58.5 and 51.2%, respectively. LWS (wheat straw acetosolv lignin) is the sample with higher cellulose and hemicellulose content but lower lignin content (Table 2). It is known that the pyrolysis of polysaccharides occurs at lower temperatures than lignin and at higher extents in this temperature interval [43]. That is why this sample showed the highest mass loss and the lowest char content (36.6%) among all samples. On the other hand, LE and LC presented a close Tpeak around 177 °C and associated mass losses of 52.9 and 55.7%, respectively. In all samples, this process was endothermic, according to DSC curves (Figure 1C).

In the case of LEB, which was the most thermally stable sample, it is the only one whose main thermal decomposition occurs in two stages. The second stage for LEB occurs in the range 103–218 °C (Tpeak 185 °C), followed by a third stage (220–457 °C, Tpeak 257 °C). The associated mass loss for both processes was 18.2 and 32.9%, respectively, for a total loss of 51.1%. It should be noted that LE and LEB were extracted from the same raw material (Eucalyptus globulus) but using different methods, which explains their dissimilar chemical structure and, therefore, their thermal stability. In addition, LEB is the sample with the highest molecular weight, which could also be the reason for its higher thermal stability. The residual char at 600 °C for all samples ranges from 36.6–40.8%, which is in the same range (31–46%) reported for lignin from other sources [46].

It is known that lignin decomposition is a complex process due to its macromolecular nature, including aromatic rings and branches and methoxyl groups. For acetosolv samples (Lignin LE, LP, and LWS), there are also acetyl groups [43]. Previous studies on lignin pyrolysis found several evolving gases (CO_2_, CO, C_2_H_4_, CH_4_, and H_2_O) during the process due to lignin dehydration, demethoxylation, C–C bond breaking, and internal rearrangements [44,47]. It is also known that different –OH groups could have different thermal stability [48], which may also be responsible for differences found in samples.

#### 3.1.6. FTIR Analysis

**Figure 2:**
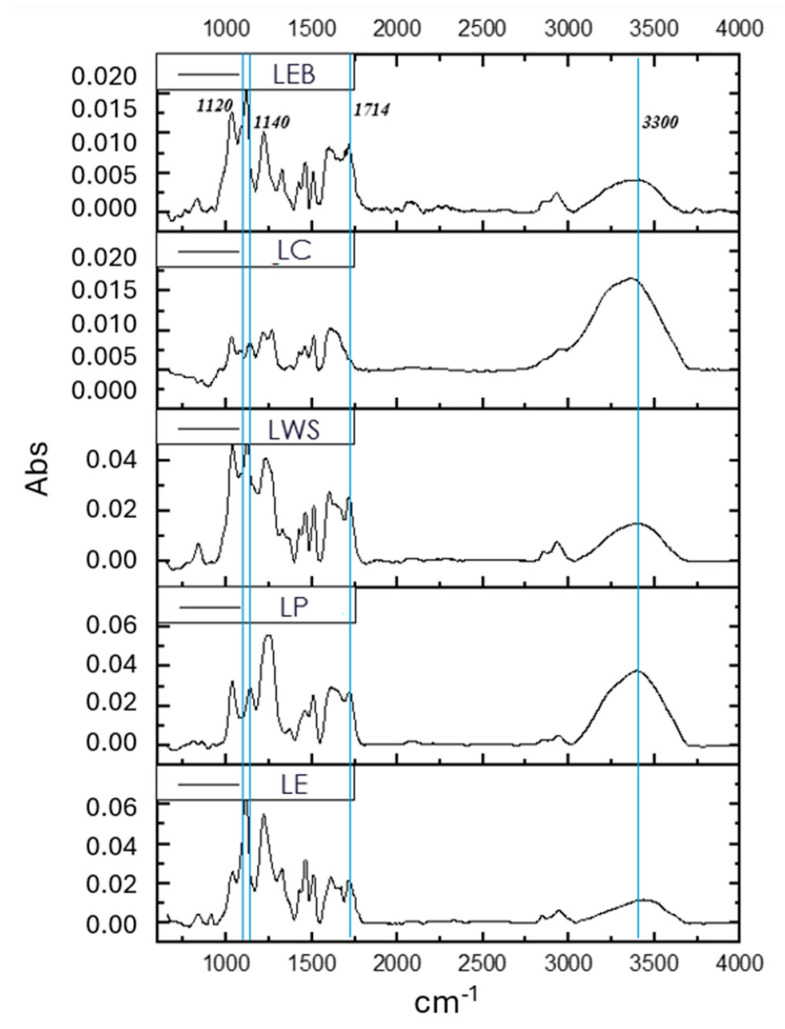
FTIR spectra of different lignin samples.

The FTIR spectra obtained from lignin samples are presented in Figure 2 and show the functional groups of this macromolecule. A broad band at 3300 cm^−1^ corresponds of OH groups of lignin. The most significant variation among the samples is the absence of a signal at 1714 cm^−1^ in LC, indicating the absence of unconjugated ketone groups. In the other samples, this band can be attributed to the presence of acetyl groups (–COCH_3_) in lignin, produced during the acetosolv and organosolv processes. The G-type lignin (LP and LC) shows bands at 1140 cm^−1^, which are derived from softwood, while the band for GS-type lignin (LE, LWS, and LEB) appears at 1120 cm^−1^ and is characteristic of hardwood, wheat straw, and hardwood bark lignins, respectively. These results are consistent with the NMR chemical characterization reported in Table 5.

### 3.2. Lignin Nanoparticle Characterization

#### 3.2.1. Dynamic Light Scattering (DLS) and Zeta Potential (ζ)

**Figure 3:**
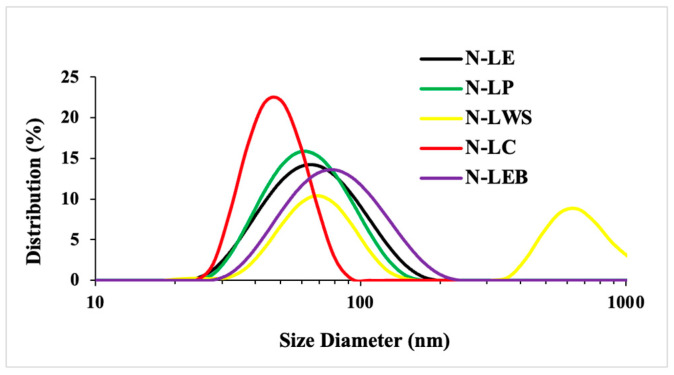
Size distribution of LNPs, concentration 1%^w^/_v_.

The determination of particle size with DLS was carried out using a solution of LNPs with an initial lignin concentration of 1%^w^/_v_ (Figure 3), which shows a well-defined population with average particle size values lower than 100 nm in nearly all LNPs, except for the N-LWS (wheat straw acetosolv). This sample showed a binodal distribution of particle size, which is probably due to the presence of modified (acetylated) and nonmodified lignin fractions. For this reason, it was decided to continue the nanoparticle characterization, excluding wheat straw acetosolv lignin.

In pursuit of higher yield, the concentration was increased to 2 and 5%^w^/_v_ (Table 7), where it was found that at concentrations of 2%^w^/_v_, the four LNP samples presented average size values of less than 100 nm, but when increased to 5%^w^/_v_, the average values exceeded 100 nm for all cases. Thus, the characterization of LNPs produced from lignin LE, LP, LC, and LEB was carried out using a concentration of 2%^w^/_v_.

**Figure 4:**
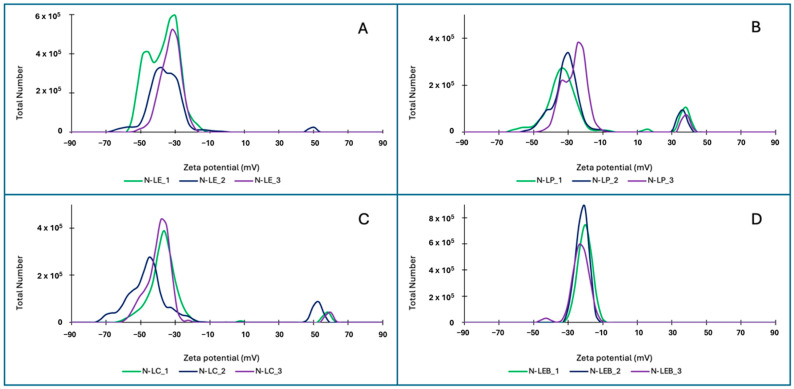
Zeta potential of LNPs, concentration 2%^w^/_v_ ((**A**): N-LE, (**B**): N-LP, (**C**): N-LC, and (**D**): N-LEB).

The zeta potential determination (Table 7) in 2%^w^/_v_ LNP from the four selected lignins showed a predominant signal in the negative sector with values ranging from −41.5 to −23.5 mV, indicating a stable colloidal system of the LNP suspensions, which corroborates the stability visually observed. However, it is worth mentioning that the LNPs from lignin LEB are the only ones that do not show any signal in the positive sector (Figure 4). In the case of LNP from lignin LE, only one of its triplicates shows a positive value. In the other LNPs from lignin LP and LC, each triplicate shows a signal of lower intensity on the positive side, which may be attributed to the presence of functional groups on the surface of the LNP that could ionize positively.

#### 3.2.2. Transmission Electron Microscopy (TEM) and Particle Size Analysis (DSL)

**Figure 5:**
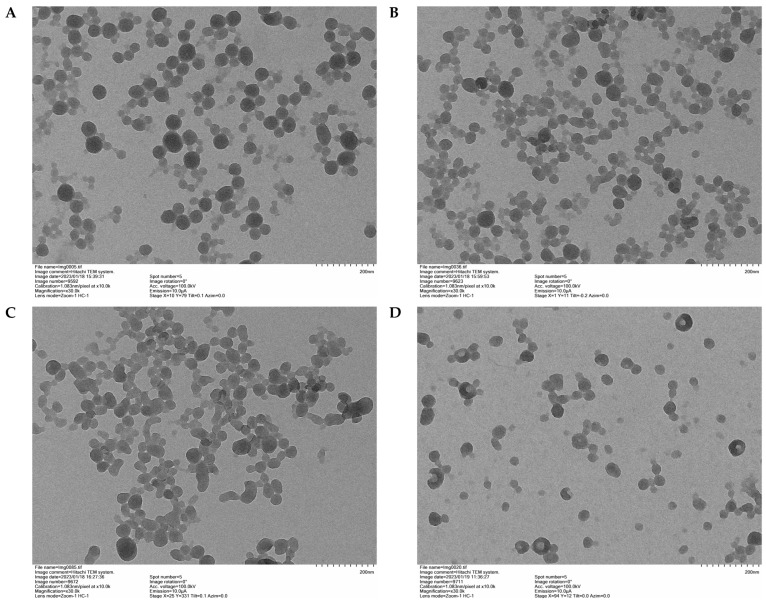
TEM analysis of LNP at a magnification ×30.0K. ((**A**): N-LE; (**B**): N-LP; (**C**): N-LC, and (**D**): N-LEB).

The TEM images (Figure 5) of the four LNPs with a 2%^w^/_v_ concentration show a similar structure with spherical particles, which corroborates the theory of the method by promoting the formation of spheres thanks to the hydrophobic and hydrophilic interactions of lignin to reduce the contact area with the precipitating agent (water). It is observed that the LNPs of N-LEB present a greater dispersion.

**Figure 6:**
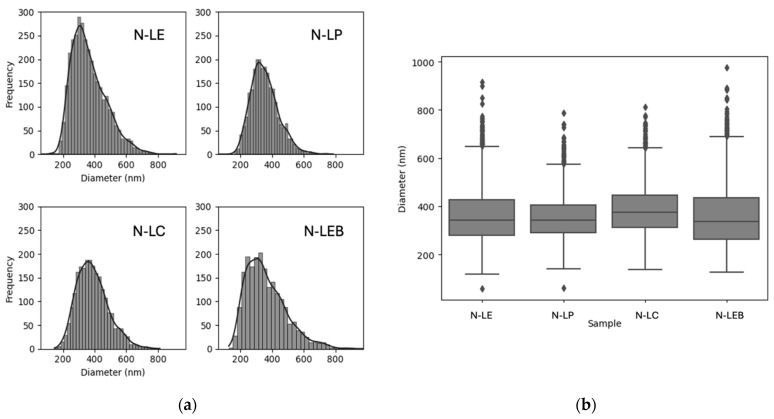
Histogram visualization of LNP particle sizes (**a**) and boxplot (**b**).

Table 8 presents mean values and standard deviations (SDs) for particle diameters across samples of LNPs (N-LE, N-LP, N-LC, and N-LEB), highlighting a notable consistency in mean particle sizes yet with variability in size uniformity, as indicated by the SDs. These numerical data are visually represented in Figure 6: the histogram in part (a) reveals the shape of the distribution, further confirming the similarity in the particle size distributions across the samples. Additionally, the boxplot in part (a) displays boxes of similar dimensions, suggesting comparable distributions among the samples.

#### 3.2.3. Determination of In Vitro Antioxidant Activity

**Figure 7:**
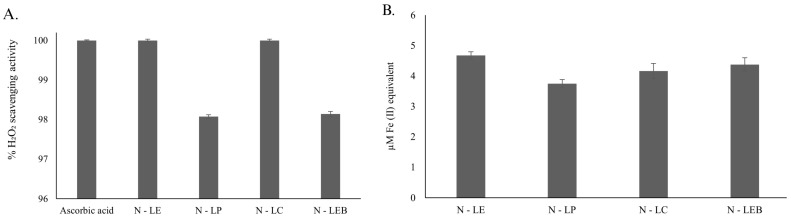
In vitro antioxidant capability of the LNPs, N-LE, N-LP, N-LC, and N-LEB (0.19% solid concentration): (**A**) H_2_O_2_-mediated free radical scavenging activity, and (**B**) ferric-reducing antioxidant power (FRAP) assay.

The remarkable absorption capacity and non-toxicity that lignin nanoparticles could present would allow them to be appropriate vehicles for drug molecules. Lignin nanoparticles have potential applications in the biomedical and environmental fields [49]. Modulation of reactive oxygen species (ROSs) levels, or ROS scavenging, is the basis of biomedical applications, as ROSs are generated in animal cells and different tissues due to aerobic metabolism. To attain stability, they oxidize other compounds [35]. The damage caused by these free radicals during oxidative stress adversely affects the biological system. Such damage is frequently linked to degenerative conditions and disorders such as cancer, cardiovascular diseases, and aging [50,51]. Substantial evidence shows that natural bioactive compounds have the ability to neutralize and stabilize ROSs [52,53]. The results of the in vitro antioxidant properties of the lignin nanoparticles are represented in Figure 7. The studied lignin nanoparticles have shown strong maximum peroxide radical scavenging activity of 100% in comparison to standard ascorbic acid.

Each of the samples studied demonstrated 100% of the H_2_O_2_-mediated oxygen-derived free radical scavenging activity. Among the lowest are N-LP and N-LEB, which gave around 98% (Figure 7A). The H_2_O_2_ scavenging activity increased by approximately 2% for samples N-LE and N-LC. Additionally, a ferric-reducing antioxidant power (FRAP) assay was used (Figure 7B) to determine the ferric ion-reducing capacity of our study samples. A higher concentration of 4.6 μM Fe(II) equivalents for metal chelation was observed in the N-LE sample. Then, sample N-LEB gave a concentration of 4.3 μM of Fe(II) iron equivalents, followed by LNP4 with 4.1 μM of Fe(II) iron equivalents, and finally, sample N-LP, which showed a concentration of 3.7 μM of Fe(II) iron equivalents, this being the lowest concentration. Thus, the lignin nanoparticles also demonstrated strong metal chelation activity. Lignins are a rich source of antioxidants, and their various phenolic functional groups are responsible for their free radical scavenging and metal ion chelation properties [54]. Lignin nanoparticles are more stable than natural lignin and show increased antioxidant potential due to their size-dependent behavior, as reported earlier by Trevisan and Rezende [32]. In our current work, the size of the lignin nanoparticle might influence its antioxidant potential.

## 4. Conclusions

The study enabled the production of LNPs using five different types of lignin from various origins and fractionation methods, including acetosolv, organosolv, and Kraft, which were thoroughly characterized. The preparation method involved precipitation with an antisolvent, using 90% acetone as the solvent and 100% water as the antisolvent to promote the formation of spherical lignin nanoparticles, as corroborated by TEM images. This method proved to be a highly convenient alternative for production due to its use of low-cost solvents, minimal environmental impact, and simple, efficient preparation with high reproducibility. Various initial lignin concentrations (1, 2, and 5%) were utilized to produce LNPs, resulting in particle sizes smaller than 100 nm in the N-LE, N-LP, N-LC, and N-LEB samples at 1% and 2% concentrations. However, the N-LWS sample, derived from wheat straw acetosolv lignin, exhibited more than one population size and an unstable solution, likely due to its low purity and high polysaccharide content. Consequently, the characterization of the four LNPs with nanometric values was conducted at a 2% concentration, yielding colloidal solutions confirmed by their zêta potential values ranging between −23.5 and −41.5 mV. Thus, lignin purity is identified as one of the most critical parameters for obtaining spherical LNPs with particle sizes lower than 100 nm and achieving more stable colloidal suspensions, highlighting the significance of the extraction process and raw material.

This work presents a significant innovation by demonstrating that it is feasible to produce stable, nanometric-sized spherical LNPs from diverse lignin sources. These LNPs offer vast potential for use as reinforcement materials due to their inherent properties, including reinforcing capability, hydrophilicity, and UV absorption, and their uses in flame retardancy, anticorrosive metal protection, and medical and drug delivery systems. Moreover, the innovation lies in the ability to create these spherical nanoparticles through a cost-effective and environmentally friendly method, enhancing their applicability in various industrial applications. The morphological advantage of spherical nanoparticles further contributes to their effectiveness when incorporated into different mixtures, making this study a new contribution to the field of lignin-based nanomaterials.

## Figures and Tables

**Table 1:** Sample Codes.

Sample	Lignin Code	LNPs Code
Lignin acetosolv *Eucalyptus globulus* (Acetic acid 87%, 1:5 solid/liquid, 160 °C for 2 h)	LE	N-LE
Lignin acetosolv *Pinus radiata* D. Don (Acetic acid 87%, 1:5 solid/liquid, 180 °C for 2 h)	LP	N-LP
Lignin acetosolv *Triticum* spp. (wheat straw) (Acetic acid 87%, 1:8 solid/liquid, 160 °C for 2 h)	LWS	N-LWS
Lignin Kraft commercial *Picea abies* and *Pinus sylvestris* (Stora Enso CAS 8068-05-1)	LC	N-LC
Lignin organosolv bark of *Eucalyptus globulus* (Ethanol 60%, 1:10 solid/liquid, 160 °C for 3 h)	LEB	N-LEB

**Table 2:** Polysaccharide and lignin content (purity) in samples isolated from different sources.

Sample	Lignin (%)	Cellulose (%)	Hemicellulose (%)
LE	89.6 ^c^ ± 0.1	0.06 ± 0.01	0.22 ± 0.01
LP	91.0 ^b^ ± 0.8	0.09 ± 0.01	0.99 ± 0.12
LWS	78.7 ^e^ ± 0.4	0.84 ± 0.10	4.91 ± 0.61
LC	93.6 ^a^ ± 0.6	0.12 ± 0.02	1.70 ± 0.27
LEB	82.5 ^d^ ± 0.5	0.20 ± 0.02	5.80 ± 0.76

The superscripts ^a^, ^b^, ^c^, ^d^ and ^e^ represent the statistical groups from the Tukey test. That is, two samples with the same letter do not have significant differences.

**Table 3:** DPPH Antioxidant Activity.

Sample	IC50 (mg/L)	AAI
LE	14.3	3.5
LP	41.3	1.2
LWS	44.0	1.1
LC	59.6	0.8
LEB	36.7	1.4
*p*-value	0.005	0.030

*p*-value < 0.05 (at least one value statistically deviates from the group mean). *p*-value > 0.05 (no difference from the mean within the group).

**Table 4:** Molecular weight determination of lignin from different sources by GPC.

Sample	Mw (g/mol)
LE	22,024
LP	21,979
LWS	24,303
LC	29,441
LEB	35,712
*p*-value	0.005

**Table 5:** Quantification of substructures in lignin samples.

Sample	β-*O*-4 (%)	β-5 (%)	β-β (%)	S/G (%)
LE	4.2	0	5.9	21.1
LP	9.6	10.7	2.4	0
LWS	35.6	1.9	10.4	1.2
LC	6.0	0.5	2.5	0
LEB	50.5	0	6.2	25.8

**Table 6:** Thermogravimetric analysis results of lignin from different sources.

Sample	Temperature (°C)			Mass Loss (%)	Char (%)
	Tonset	Tpeak	Tend		
LE	71	178	467	52.3	40.8
LP	72	157	431	51.2	40.1
LWC	108	140	451	58.5	36.6
LC	104	176	453	55.7	38.6
LEB	103	185	218	18.1	39.4
	220	257	457	34.9	

**Table 7:** Particle size distribution and zeta potential measurements of lignin nanoparticles.

Sample	Size (nm) LNP 5%^w^/_v_	Size (nm) LNP 2%^w^/_v_	Polydispersity Index (PDI) LNP 2%^w^/_v_	Zeta Potential (ζ) (mV) LNP 2%^w^/_v_
N-LE	113.9 ± 2.4	90.7 ^a^ ± 0.1	0.10 ^b^ ± 0.02	−35.0 ^b^ ± 1.6
				+50.3 ± 0.0
N-LP	123.5 ± 0.11	77.0 ^b^ ± 0.10	0.10 ^b^ ± 0.03	−30.7 ^b^ ± 2.7
				+36.8 ± 1.9
N-LC	145.9 ± 0.22	77.1 ^b^ ± 0.09	0.09 ^b^ ± 0.01	−41.5 ^c^ ± 1.9
				+56.6 ± 4.5
N-LEB	154.6 ± 7.7	88.1 ^a^ ± 1.0	0.26 ^a^ ± 0.02	−23.5 ^a^ ± 3.0

The superscripts ^a, b^ and ^c^ represent the statistical groups of the Tukey test. That is, two samples with the same letter do not have significant differences.

**Table 8:** Summary of mean diameters and SDs for LNP samples.

Sample	Mean (nm)	SD (nm)
N-LE	362.5	109.9
N-LP	353.1	88.5
N-LC	353.1	100.6
N-LEB	361.0	126.3
N-LE	362.5	109.9

## Data Availability

The original contributions presented in the study are included in the article/Supplementary Material, further inquiries can be directed to the corresponding authors.

## Supplementary Materials

The following supporting information can be downloaded at: https://www.mdpi.com/article/10.3390/polym16111610/s1, Figure S1: NMR 90-50 et 2.5-6 (ppm)-LE; Figure S2: NMR 90-50 et 2.5-6 (ppm)-LP; Figure S3: NMR 90-50 et 2.5-6 (ppm)-LWS; Figure S4: NMR 90-50 et 2.5-6 (ppm)-LC; Figure S5: NMR 90-50 et 2.5-6 (ppm)-LEB; Figure S6: NMR 140-100 et 8.5-5.5 (ppm)-LE; Figure S7: NMR 140-100 et 8.5-5.5 (ppm)-LP; Figure S8: NMR 140-100 et 8.5-5.5 (ppm)-LWS; Figure S9: NMR 140-100 et 8.5-5.5 (ppm)-LC; Figure S10: NMR 140-100 et 8.5-5.5 (ppm)-LEB.
