# Supplementary material for: Preparation and Characterization of Lignin Nanoparticles from Different Plant Sources
Source: Polymers (Basel). 2024 Jun 6;16(11):1610. doi: 10.3390/polym16111610 (PMC11174508; doi:10.3390/polym16111610)

Article

Preparation and characterization of lignin nanoparticles from different plant sources

Isidora Ortega-Sanhueza [1,*], Victor Girard [2], Isabelle Ziegler-Devin [2], Hubert Chapuis [2], Nicolas Brosse [2], Francisca Valenzuela [3], Aparna Banerjee [3], Cecilia Fuentalba [4,5], Gustavo Cabrera-Barjas [6], Camilo Torres [1], Alejando Méndez [1], César Segovia [7] and Miguel Pereira [8,*]

[1] Facultad de Ciencias Forestales, Universidad de Concepción, Concepción, Chile
[2] Laboratoire d'Etude et de Recherche sur le MAtériau Bois (LERMAB), Faculté des Sciences et Techniques, Université de Lorraine, Vandœuvre-lès-Nancy, France
[3] Instituto de Ciencias Aplicadas, Facultad de Ingeniería, Universidad Autónoma de Chile, Talca, 3467987, Chile
[4] Unidad de Desarrollo Tecnológico (UDT), Universidad de Concepción, Av. Cordillera 2634, Parque Industrial Coronel, P.O. Box 4051 mail 3, Concepción, Chile.
[5] Centro Nacional de Excelencia para la Industria de la Madera (CENAMAD), Pontificia Universidad Católica de Chile, Av. Vicuña Mackena, 4860, Santiago 7820436, Chile.
[6] Facultad de Ciencias para el Cuidado de la Salud, Universidad San Sebastián Campus Las Tres Pascualas, Lientur 1457, CP 4080871, Concepción, Chile.
[7] Centre d'Essais Textile Lorrain, CETELOR – Université de Lorraine. 27 rue Philippe Seguin, 88051 Epinal, France
[8] Facultad de Ingeniería, Departamento de Ingeniería Química, Universidad de Concepción, Concepción, Chile.
* Correspondence: isiortega@udec.cl (I.O.-S.); miguelpereira@udec.cl (M.P.)

Figure S1: NMR 90-50 et 2.5-6 (ppm) - LE
Figure S2: NMR 90-50 et 2.5-6 (ppm) - LP
Figure S3: NMR 90-50 et 2.5-6 (ppm) - LWS
Figure S4: NMR 90-50 et 2.5-6 (ppm) – LC
Figure S5: NMR 90-50 et 2.5-6 (ppm) - LEB
Figure S6: NMR 140-100 et 8.5-5.5 (ppm) - LE
Figure S7: NMR 140-100 et 8.5-5.5 (ppm) - LP
Figure S8: NMR 140-100 et 8.5-5.5 (ppm) - LWS
Figure S9: NMR 140-100 et 8.5-5.5 (ppm) – LC
Figure S10: NMR 140-100 et 8.5-5.5 (ppm) - LEB

90-50 et 2,5-6

S1

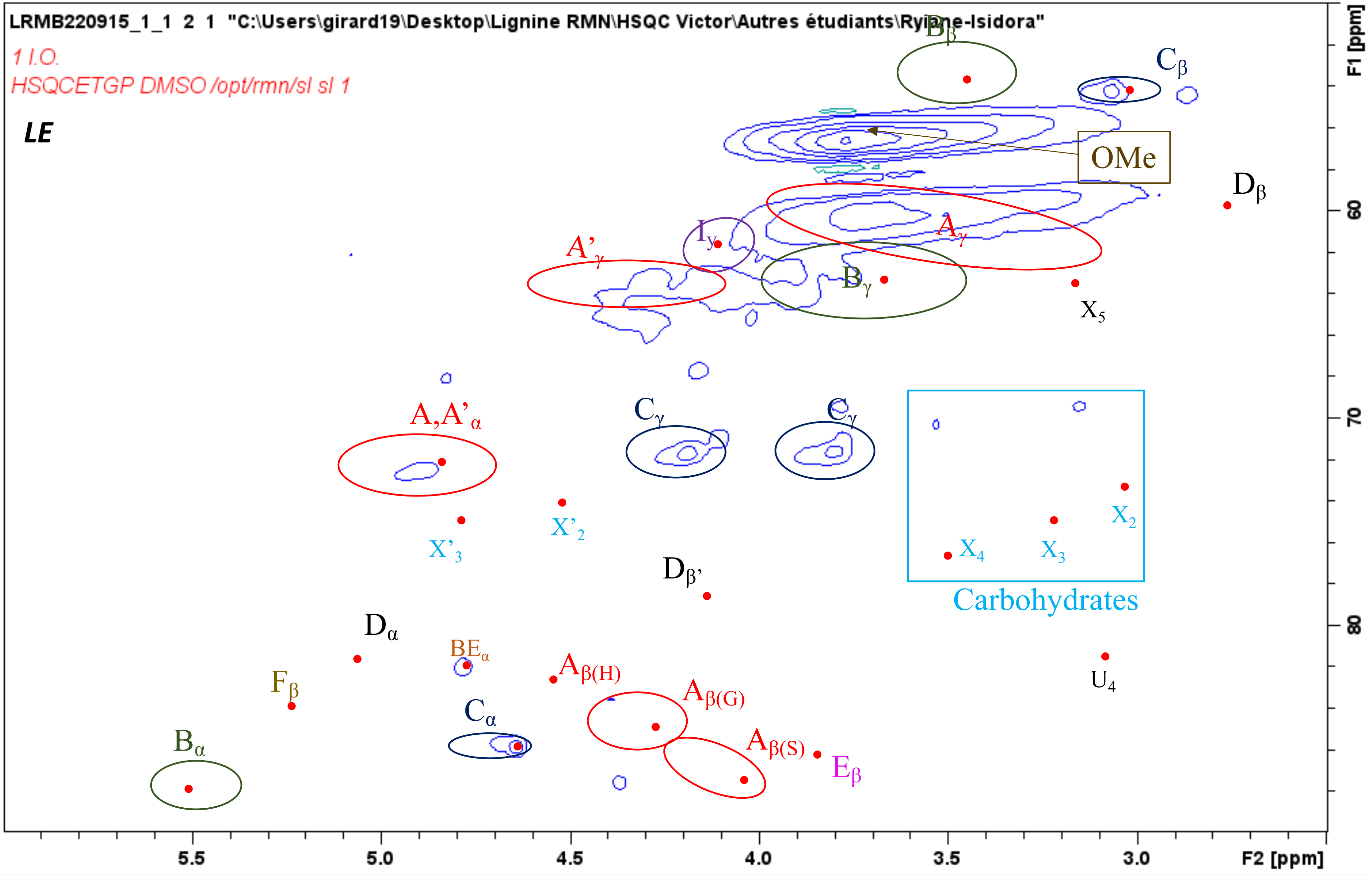

90-50 et 2,5-6

S2

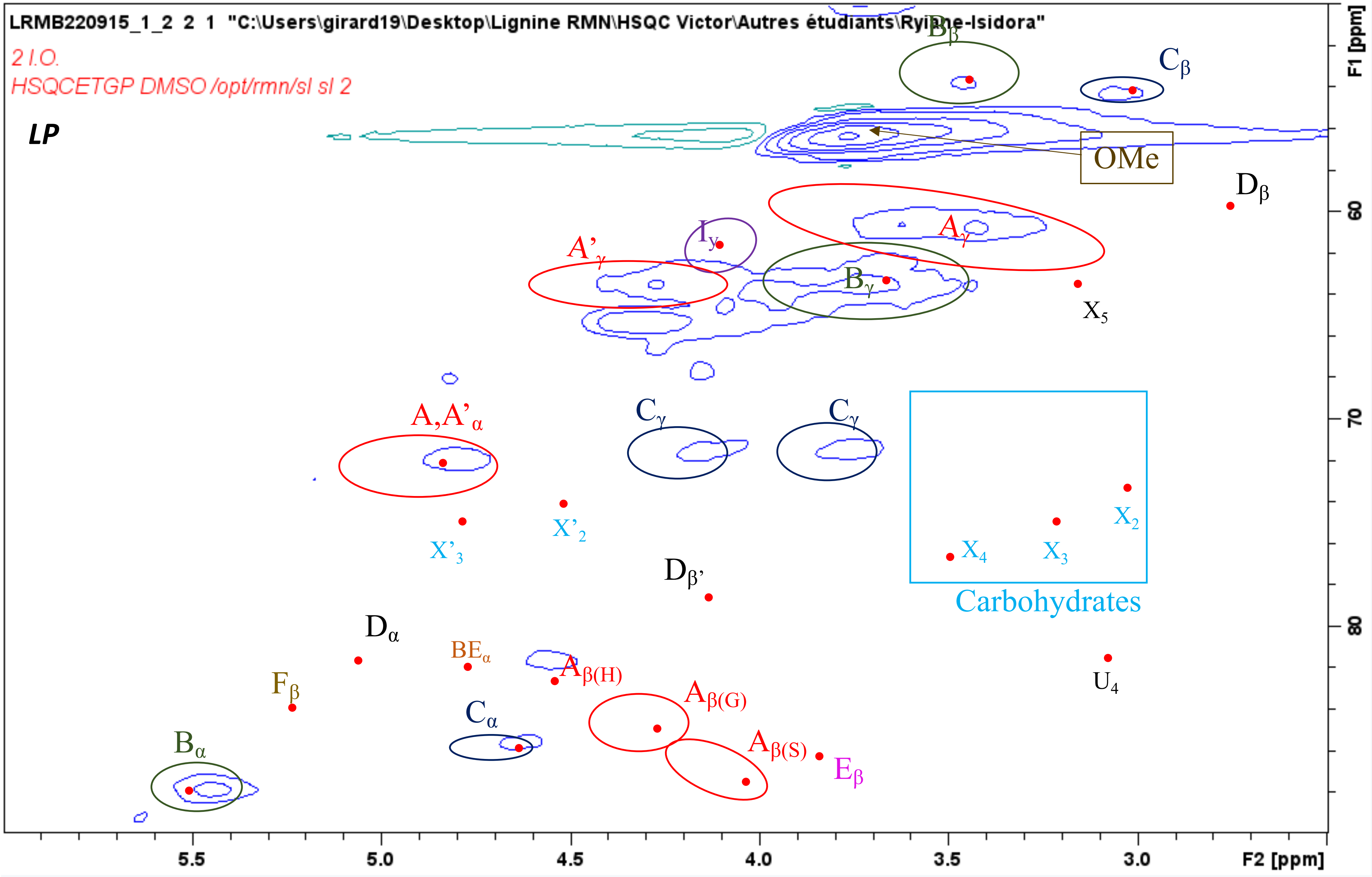

90-50 et 2,5-6

S3

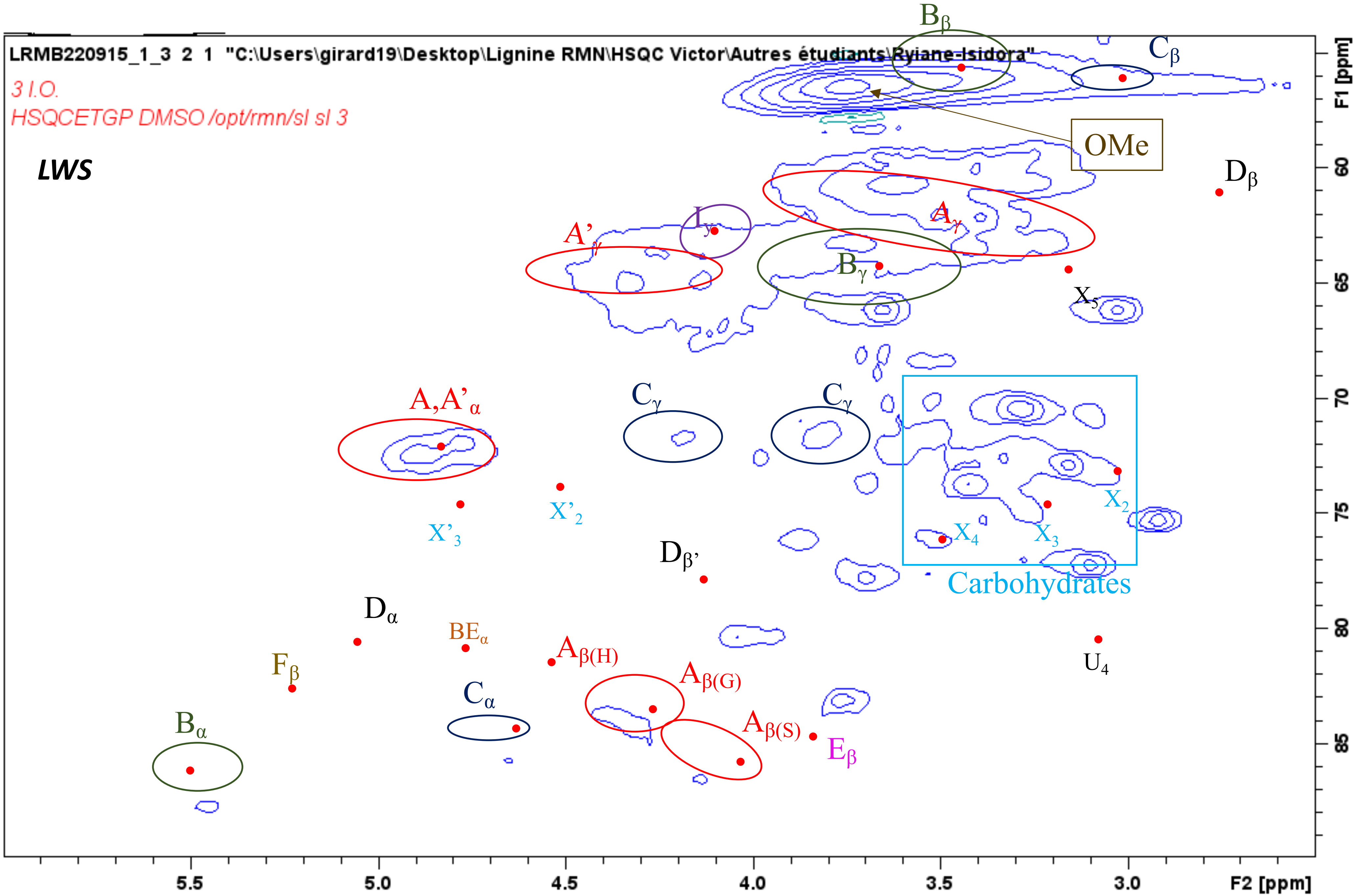

90-50 et 2,5-6

S4 ***LC***

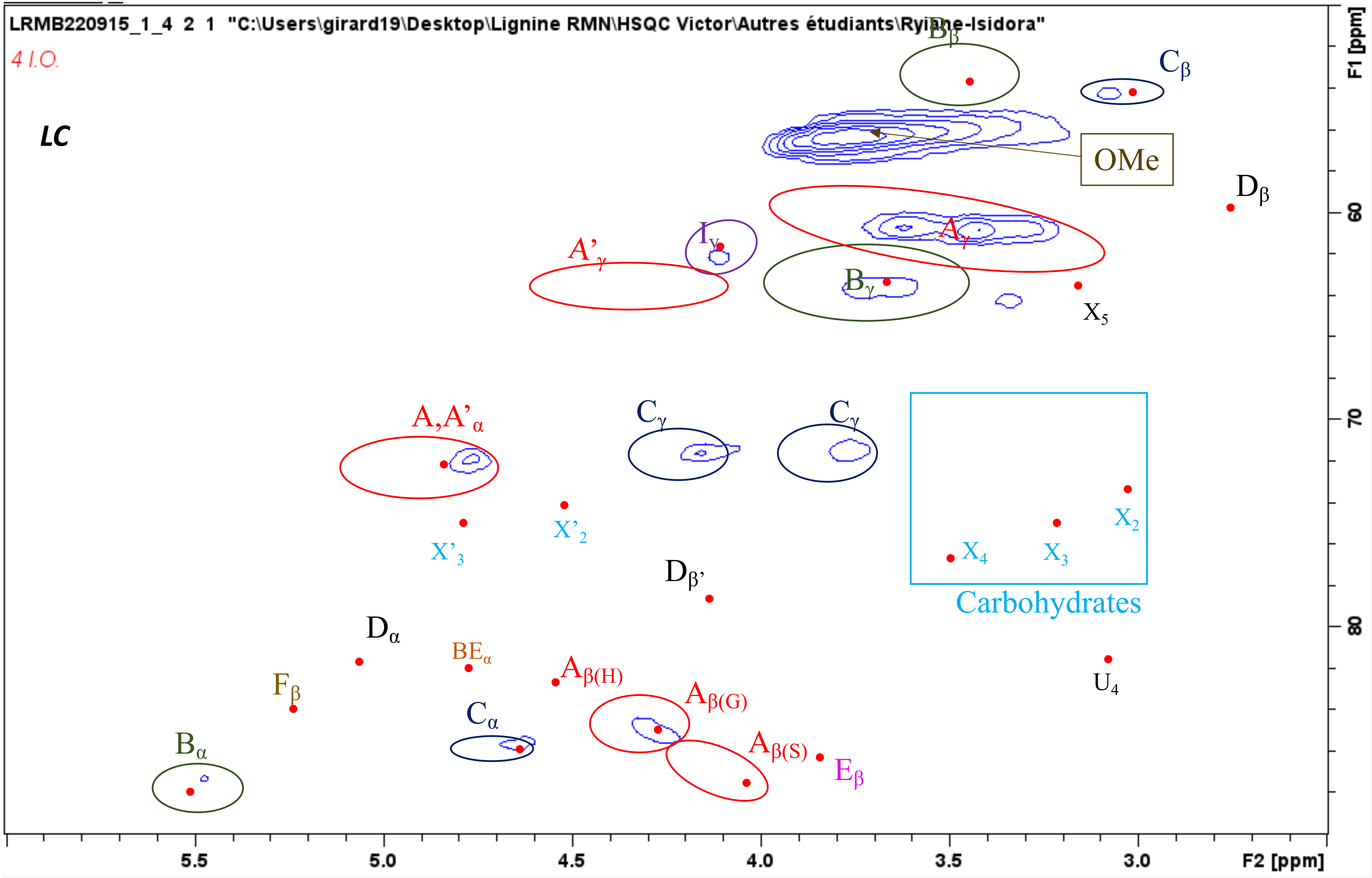

90-50 et 2,5-6

S5 ***LEB***

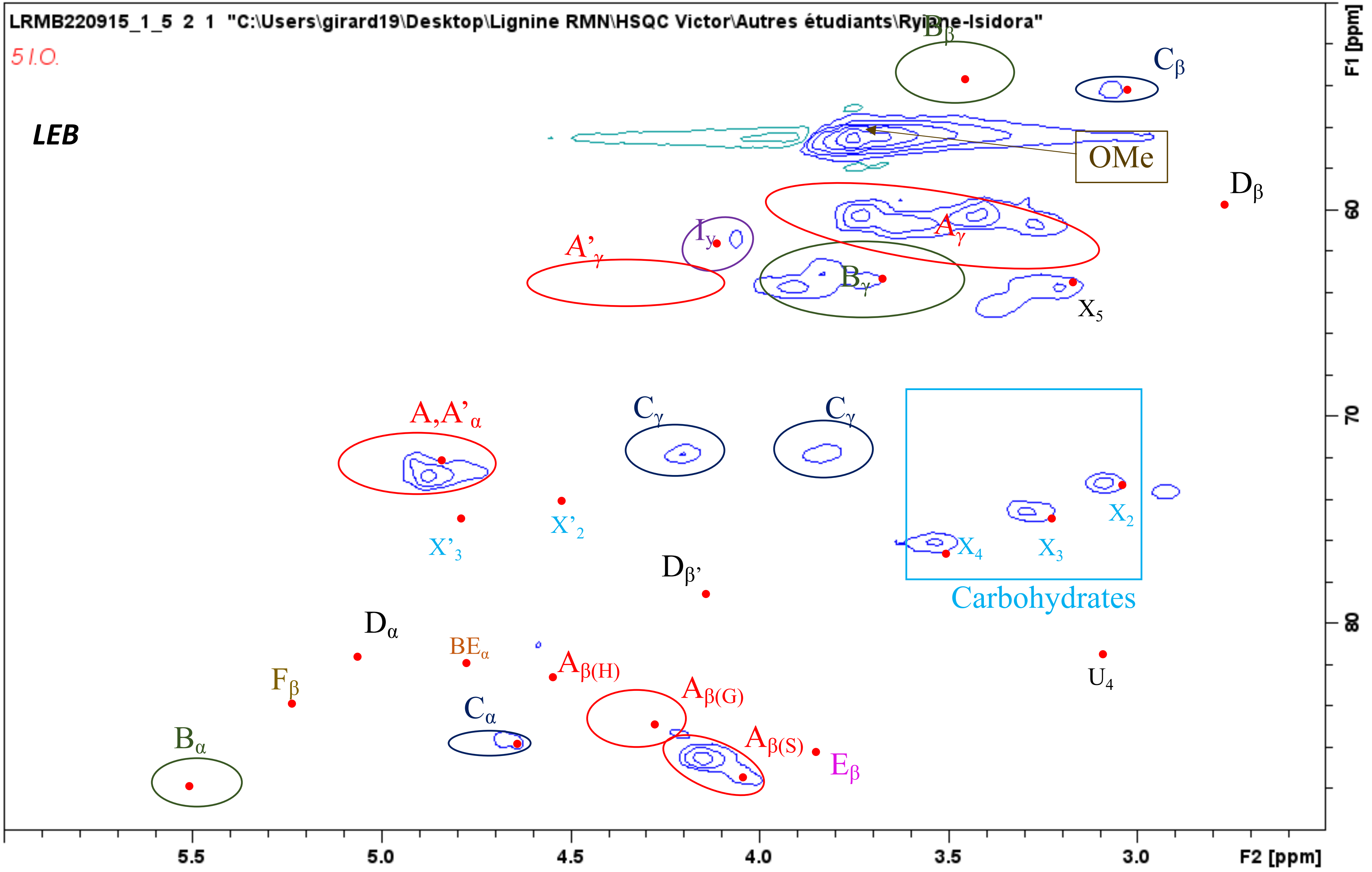

140-100 et 8,5-5,5

S6

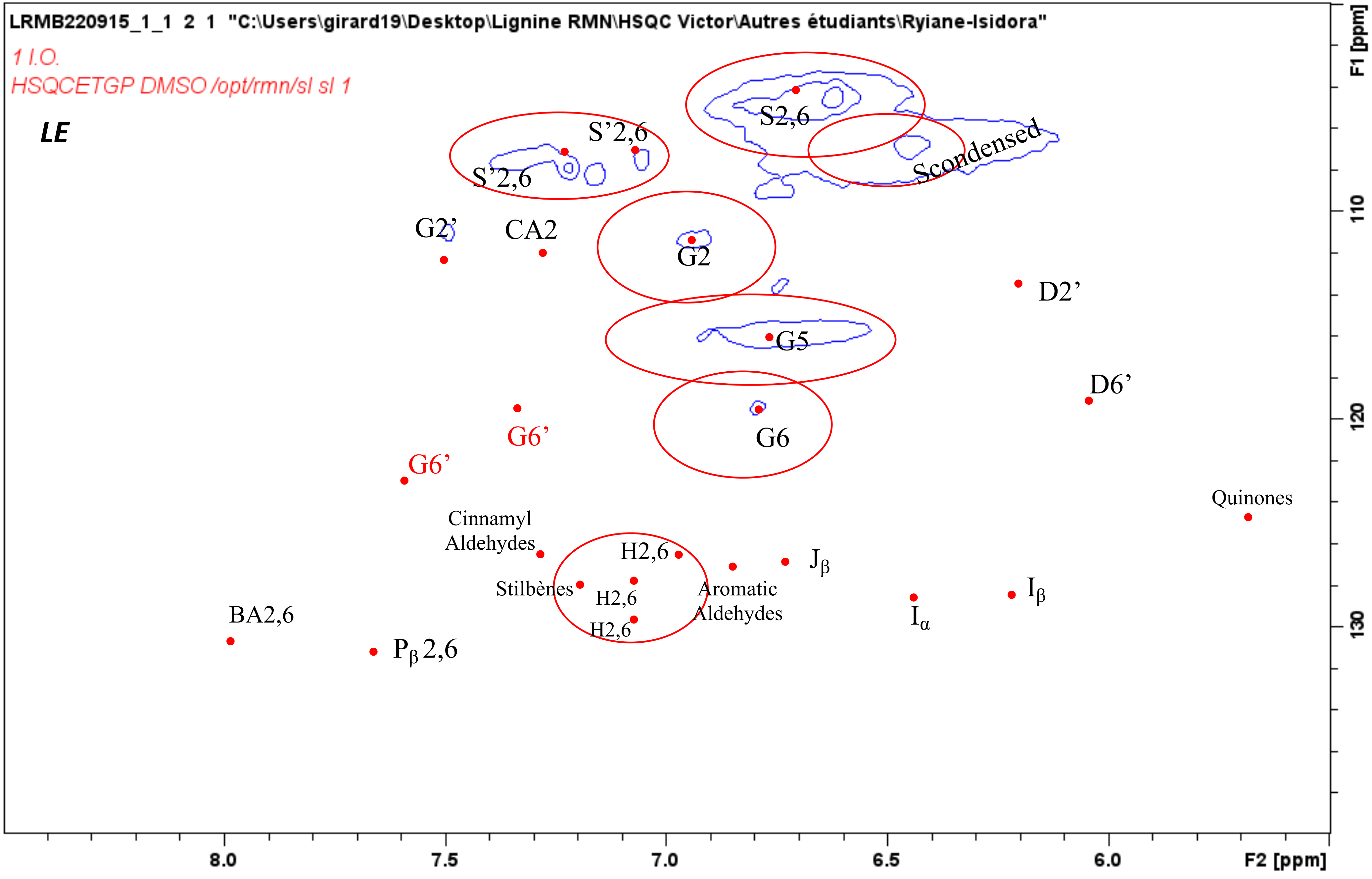

140-100 et 8,5-5,5

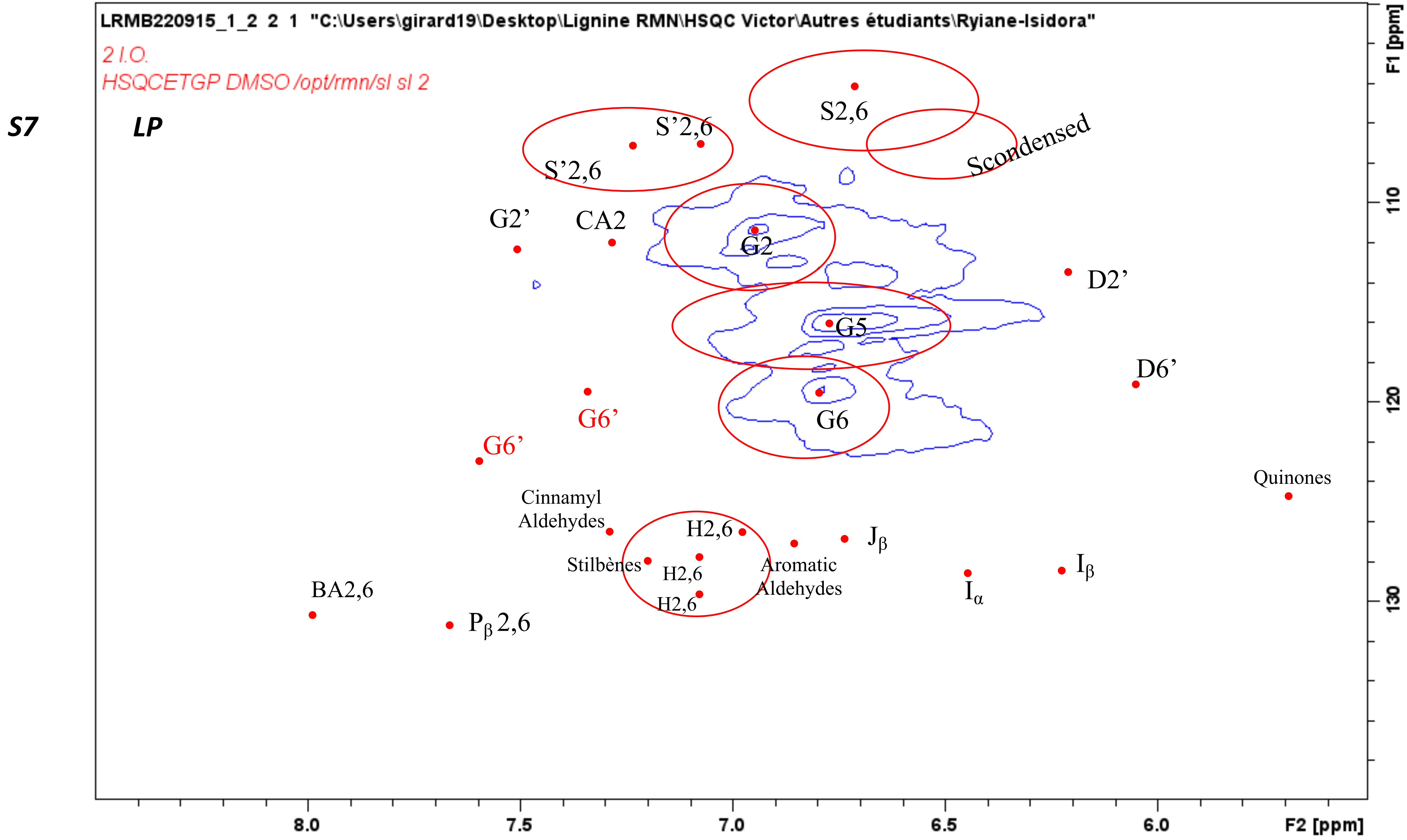

140-100 et 8,5-5,5

S8 **LWS**

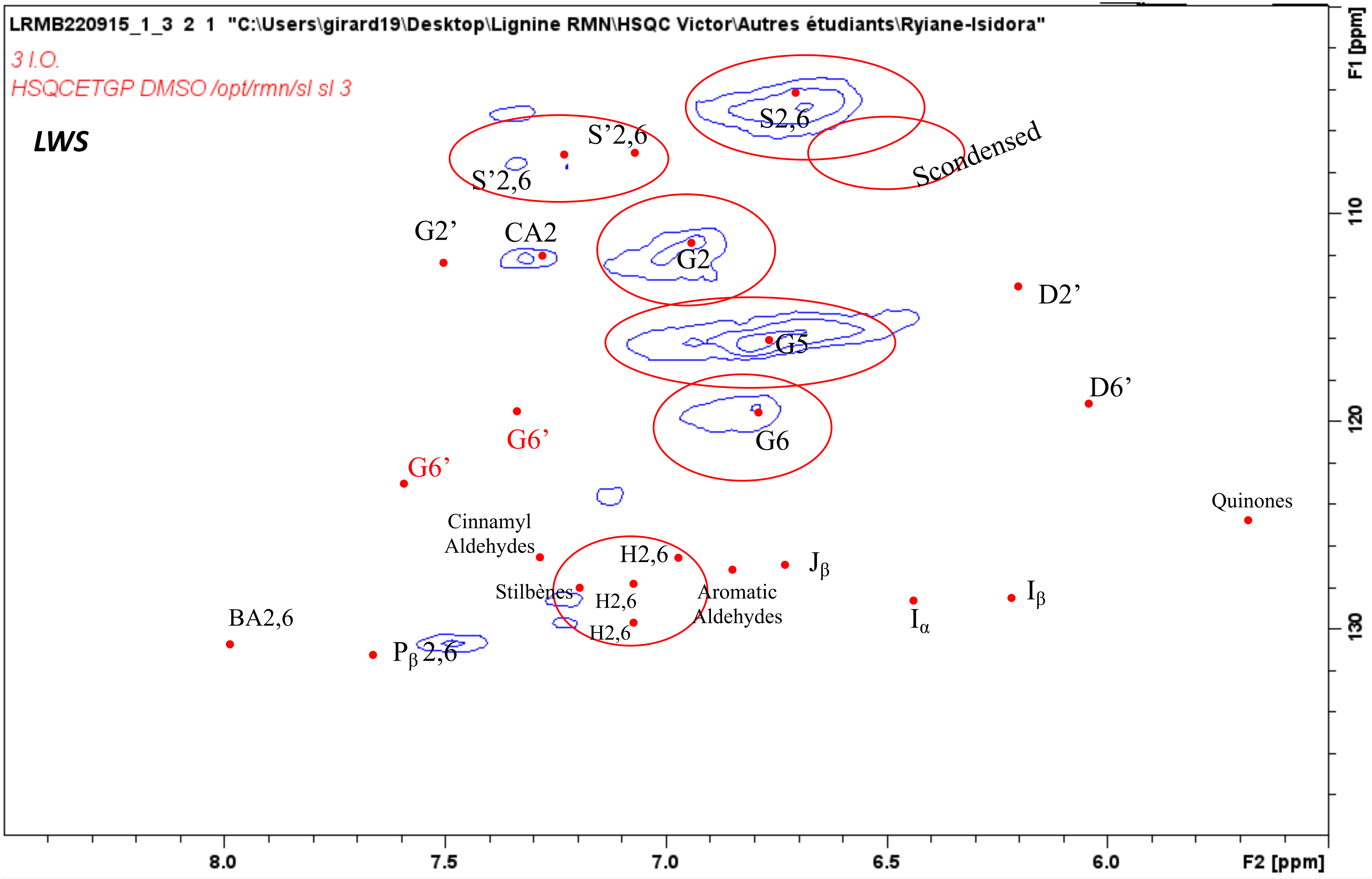

140-100 et 8,5-5,5

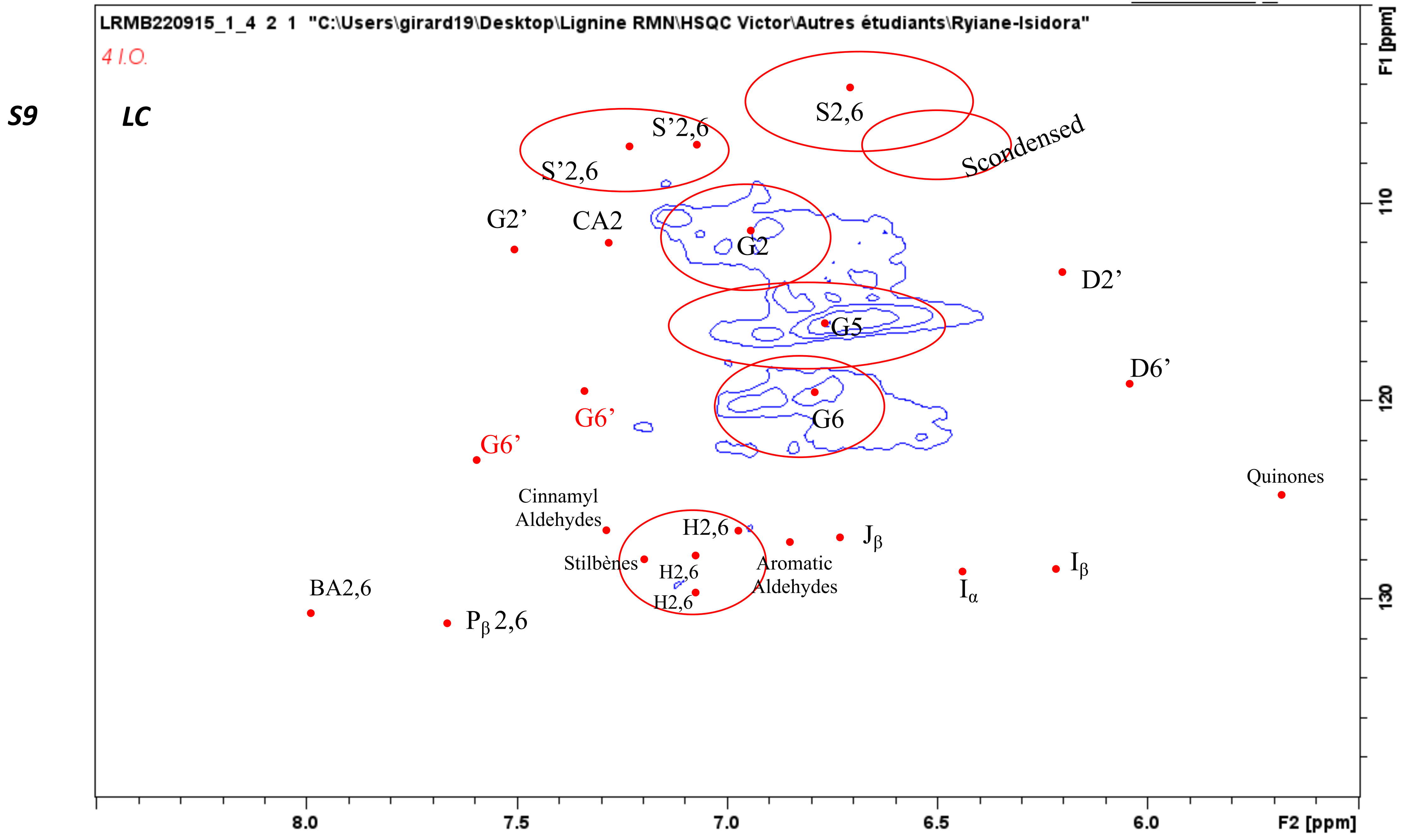

140-100 et 8,5-5,5

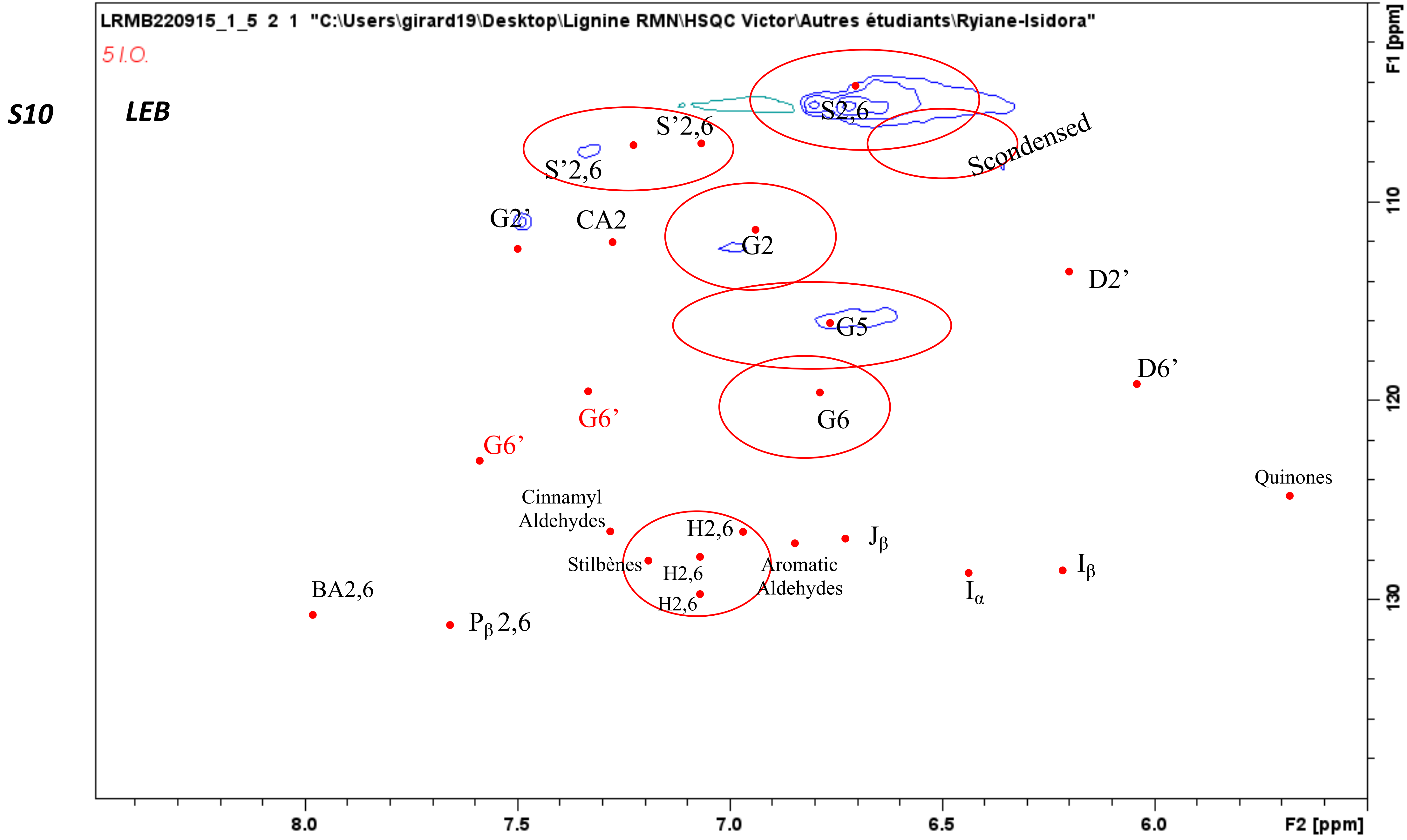

Supplement: Supplementary file 1 [file polymers-16-01610-s001.zip › polymers-3027864-supplementary.pdf]
